# Supplementary material for: Modeling glioblastoma heterogeneity as a dynamic network of cell states
Source: Mol Syst Biol. 2021 Sep 16;17(9):e10105. doi: 10.15252/msb.202010105 (PMC8444284; doi:10.15252/msb.202010105)
Supplement: Supplementary file 6 — Source Data for Figure 5 [file MSB-17-e10105-s004.zip › Figure5A_sourcedata/GSEA_3071/hallmarks_stateVI.GseaPreranked.1623742557031/HALLMARK_ALLOGRAFT_REJECTION.html]

Details for gene set HALLMARK\_ALLOGRAFT\_REJECTION[GSEA]

|  || Dataset | state63071 |
| Phenotype | NoPhenotypeAvailable |
| Upregulated in class | na\_pos |
| GeneSet | HALLMARK\_ALLOGRAFT\_REJECTION |
| Enrichment Score (ES) | 0.30507264 |
| Normalized Enrichment Score (NES) | 1.6605844 |
| Nominal p-value | 0.01746725 |
| FDR q-value | 0.042184245 |
| FWER p-Value | 0.161 |
Table: GSEA Results Summary

  

Fig 1: Enrichment plot: HALLMARK\_ALLOGRAFT\_REJECTION      
 Profile of the Running ES Score & Positions of GeneSet Members on the Rank Ordered List

  

| PROBE | GENE SYMBOL | GENE\_TITLE | RANK IN GENE LIST | RANK METRIC SCORE | RUNNING ES | CORE ENRICHMENT || 1 | IL11 |  |  | 18 | 1.044 | 0.0615 | Yes |
| 2 | INHBA |  |  | 80 | 0.781 | 0.0943 | Yes |
| 3 | NPM1 |  |  | 114 | 0.717 | 0.1308 | Yes |
| 4 | EIF3D |  |  | 152 | 0.663 | 0.1628 | Yes |
| 5 | IL18 |  |  | 239 | 0.572 | 0.1753 | Yes |
| 6 | RIPK2 |  |  | 267 | 0.555 | 0.2032 | Yes |
| 7 | EIF5A |  |  | 361 | 0.507 | 0.2096 | Yes |
| 8 | MTIF2 |  |  | 375 | 0.499 | 0.2378 | Yes |
| 9 | NME1 |  |  | 405 | 0.487 | 0.2607 | Yes |
| 10 | SOCS1 |  |  | 489 | 0.458 | 0.2669 | Yes |
| 11 | EIF3J |  |  | 664 | 0.408 | 0.2446 | Yes |
| 12 | EGFR |  |  | 669 | 0.407 | 0.2694 | Yes |
| 13 | MRPL3 |  |  | 719 | 0.392 | 0.2808 | Yes |
| 14 | BCAT1 |  |  | 791 | 0.378 | 0.2852 | Yes |
| 15 | CAPG |  |  | 851 | 0.368 | 0.2923 | Yes |
| 16 | ABCE1 |  |  | 889 | 0.362 | 0.3051 | Yes |
| 17 | CCND2 |  |  | 1064 | 0.335 | 0.2781 | No |
| 18 | IL15 |  |  | 1353 | 0.297 | 0.2171 | No |
| 19 | RPL9 |  |  | 1399 | 0.292 | 0.2232 | No |
| 20 | HIF1A |  |  | 1458 | 0.284 | 0.2252 | No |
| 21 | CSK |  |  | 1571 | 0.272 | 0.2114 | No |
| 22 | EIF3A |  |  | 1650 | 0.264 | 0.2066 | No |
| 23 | FLNA |  |  | 1737 | 0.257 | 0.1991 | No |
| 24 | LIF |  |  | 1804 | 0.250 | 0.1968 | No |
| 25 | TAPBP |  |  | 1817 | -0.252 | 0.2094 | No |
| 26 | NCK1 |  |  | 2174 | -0.309 | 0.1304 | No |
| 27 | STAT1 |  |  | 2199 | -0.314 | 0.1437 | No |
| 28 | THY1 |  |  | 2265 | -0.326 | 0.1464 | No |
| 29 | EIF4G3 |  |  | 2364 | -0.348 | 0.1414 | No |
| 30 | B2M |  |  | 2460 | -0.369 | 0.1385 | No |
| 31 | F2R |  |  | 2478 | -0.372 | 0.1574 | No |
| 32 | ST8SIA4 |  |  | 2719 | -0.430 | 0.1182 | No |
| 33 | GALNT1 |  |  | 2978 | -0.528 | 0.0803 | No |
| 34 | BRCA1 |  |  | 3335 | -0.802 | 0.0326 | No |
| 35 | TGFB2 |  |  | 3340 | -0.804 | 0.0827 | No |
Table: GSEA details [plain text format]

  

Fig 2: HALLMARK\_ALLOGRAFT\_REJECTION: Random ES distribution      
 Gene set null distribution of ES for **HALLMARK\_ALLOGRAFT\_REJECTION**
